# Variation of DNA Methylation in Newborns Associated with Exhaled Carbon Monoxide during Pregnancy

**DOI:** 10.3390/ijerph18041597

**Published:** 2021-02-08

**Authors:** Ediane De Queiroz Andrade, Gabriela Martins Costa Gomes, Adam Collison, Jane Grehan, Vanessa E. Murphy, Peter Gibson, Joerg Mattes, Wilfried Karmaus

**Affiliations:** 1 School of Medicine and Public Health, University of Newcastle, Newcastle, NSW 2308, Australia; ediane.dequeirozandrade@uon.edu.au (E.D.Q.A.); gabriela.martinscostagomes@uon.edu.au (G.M.C.G.); jane.grehan@newcastle.edu.au (J.G.); vanessa.murphy@newcastle.edu.au (V.E.M.); joerg.mattes@newcastle.edu.au (J.M.); 2 Priority Research Centre GrowUpWell, Hunter Medical Research Institute, University of Newcastle, Newcastle, NSW 2308, Australia; 3 Priority Research Centre Healthy Lungs, Hunter Medical Research Institute, University of Newcastle, Newcastle, NSW 2308, Australia; peter.gibson@newcastle.edu.au; 4 Respiratory & Sleep Medicine Department, John Hunter Hospital, Newcastle, NSW 2305, Australia; 5 Paediatric Respiratory & Sleep Medicine Department, John Hunter Children’s Hospital, Newcastle, NSW 2305, Australia; 6 Division of Epidemiology, Biostatistics, and Environmental Health Science, School of Public Health, The University of Memphis, Memphis, TN 38152, USA

**Keywords:** maternal exposure, tobacco use, epigenetic epidemiology, fetal programming, epigenome-wide association studies

## Abstract

Fetal exposure to tobacco smoke is an adverse risk factor for newborns. A plausible mechanism of how this exposure may negatively impact long term health is differential methylation of deoxyribonucleic acid (DNAm) and its relation to birth weight. We examined whether self-reported gestational smoking status and maternal exhaled carbon monoxide (eCO) during early pregnancy were associated with methylation of cytosine by guanines (CpG) sites that themselves predicted birth weight. We focused first on CpGs associated with maternal smoking, and secondly, among these, on CpGs related to birth weight found in another cohort. Then in 94 newborns from the Breathing for Life Trial (BLT) DNAm levels in cord blood were determined using Infinium Methylation EPIC BeadChip measuring >850K CpGs. We regressed CpGs on eCO and tested via mediation analysis whether CpGs link eCO to birth weight. Nine smoking related CpG sites were significantly associated with birth weight. Among these nine CpGs the methylation of cg02264407 on the *LMO7* gene was statistically significant and linked with eCO measurements. eCO greater than six ppm showed a 2.3% decrease in infant DNAm (*p* = 0.035) on the *LMO7* gene. A 1% decrease in methylation at this site resulted in decreased birth weight by 44.8 g (*p* = 0.003). None of the nine CpGs tested was associated with self-reported smoking. This is the first study to report potential mediation of DNA methylation, linking eCO measurements during early pregnancy with birth weight.

## 1. Introduction

Fetal exposure to tobacco smoke throughout pregnancy is prevalent and a preventable risk for child morbidity and mortality [1,2]. Despite efforts of international health organisations to raise awareness for risk to the developing fetus, at least 15% to 20% of mothers do not quit smoking during pregnancy [3]. Previous research has described associations between in utero [4] tobacco smoke exposure with severe neonatal outcomes such as prematurity [5], stillbirth [6], congenital anomalies [7], low birth weight [8], and neonatal mortality [8]. One of the most widely reported effects is on birth weight [9,10,11].

Birth weight is an informative indicator of pregnancy outcome and neonatal health. Both low and high birth weight categories have been linked to adverse health outcomes later in life [12,13,14,15]. Numerous epidemiological studies have focused on understanding the early life determinants of changes on the structural and physiological metabolic functions of the fetus [16,17,18,19]. Barker et al., who first observed these associations, hypothesised that programmed changes during this critical period of development predispose the fetus to certain postnatal diseases [20].

A plausible mechanism through which exposure to adverse intrauterine conditions may negatively impact long term health is through modulation of deoxyribonucleic acid (DNA) methylation. It has been suggested that the effects of maternal factors during pregnancy on birth weight are exerted through differential methylation of DNA in offspring [21]. A meta-analysis of epigenome-wide association studies (EWAS) on birth weight and neonatal DNA methylation (DNAm) reported that 914 cytosine-phosphate-guanine sites (CpGs) are differentially methylated in association with birth weight [22]. However, due to concurrent measurements of birth weight and DNAm, it remains unclear whether differentially methylated sites represent the effects of lower birth weight or are risk factors for differences in birth weight.

DNAm has a vital role in repressing gene expression by blocking promoters at which activating transcription factors could bind, thereby controlling cell differentiation and embryonic development [23]. Sutter et al. suggested that maternal smoking dysregulates placental methylation in a CpG site-specific way that correlates with modifications in gene expression along signature pathways, leading to a significant reduction in newborn birth weight among the newborn of smokers [24]. Another mode of action of DNAm is regulation of alternative splicing resulting in gene expression of different transcripts [25].

Although previous studies [26,27] have investigated whether DNAm mediates maternal smoking and birth weight, their approach depended on self-reported smoking status. Self-reports are a possible source of information bias and may underestimate associations with maternal smoking [28].

To the best of our knowledge, no study has explored whether the mediation of DNAm after carbon monoxide exposure in early pregnancy would affect birth weight, direct and indirectly. This study aims to investigate CpG methylation in cord blood samples with self-reported maternal smoking during pregnancy and with exhaled carbon monoxide (eCO) as a biochemical marker for current cigarette exposure. To reduce the number of CpG sites to be tested we focused on DNA methylation sites related to smoking as reported by Joubert et al. [29] based on a meta-analysis of multiple studies. Then, to further concentrate, we used the Isle of Wight Birth Cohort (IoW birth cohort) [30] to identify which of the smoking-related CpGs are also associated with birth weight. Second, we analysed a subgroup of 94 infants by investigating DNAm in cord blood samples. We tested associations of known smoking-related CpG sites with maternal smoking (questionnaire and eCO in specific time-windows). Third, we investigated whether there is a pathway from smoking exposure via DNA methylation (as mediator) to differential birth weights.

## 2. Materials and Methods

### 2.1. Study Design

This investigation focuses on a subsample of infants from the randomised controlled trial (RCT), the Breathing for Life Trial (BLT) [31]. BLT is a multi-center RCT, which recruited pregnant women with current doctor-diagnosed asthma from public hospital antenatal clinics in Newcastle, Sydney, Canberra, and Brisbane. Women were randomised between 12- and 23-weeks’ gestation to asthma management guided by fractional exhaled nitric oxide (FeNO) or to usual clinical care. Women <18 years of age or with drug/alcohol dependence were excluded. In a sub-group of the participants, cord blood samples were collected after delivery. This study was approved by the Hunter New England Human Research Ethics Committee (Reference Number 12/10/17/3.04, NSW HREC Reference No: HREC/12/ HNE/357), Australia, and participation was based on written informed consent.

At enrolment, between 12 and 23 weeks gestation, information was collected about sociodemographic characteristics and lifestyle factors, such as self-reported maternal smoking, maternal age, ethnicity, level of education, parity, health status, height, weight, and exhaled carbon monoxide (eCO) (piCO Smokerlyzer Breath CO Monitor, Bedfont, UK). Postcodes were collected at baseline and used for socioeconomic status within state or territory based on the Socioeconomic Index for Areas (SEIFA) percentiles [32]. For those randomised to usual care, only one study visit was provided. Participants randomised to the intervention group had monthly visits until delivery.

After delivery, trained staff blinded to the intervention group extracted information from medical records on gestational age at birth, birth weight, birth length, type of delivery, maternal and neonatal complications.

### 2.2. Cord Blood Collection

Cord blood samples from BLT participants were collected at John Hunter Hospital (New South Wales—Australia) immediately after birth by needle puncture of the umbilical vein after the umbilical cord was detached from the infant. Samples were transferred into EDTA tubes to be processed within six hours.

### 2.3. Flow Cytometry Staining

Cord blood cells were stained in whole blood and subsets were predefined based on specific surface markers as follow: Eosinophils (CD45^+^, CD193^+^, CD16^−^), Neutrophils (CD45^+^, CD193^−^, CD16^+^), B cells (CD14^−^, CD3^−^, CD19^+^), Natural Killer (NK) cells (CD14^−^, CD3^−^, CD56^+^, CD16^+^), lymphocytes TCD4 cells (CD3^+^, αβT-cell receptor (TCR)^+^, CD4^+^, CD25^+^, CD127^+^), lymphocytes TCD8 cells (CD3^+^, αβTCR^+^, CD8^+^, CD25^+^, CD127^+^) and Monocytes (CD3^−^, CD19^−^, CD56^−^, CD14^+^, CD16^+^, HLA-DR^+^). After 30 min of incubation, red blood cells were lysed using BD FACS™ Lysing Solution and washed. Samples were stored at 4 °C until acquisition of LSRFortessa X-20 flow cytometer (BD Biosciences, San Diego, CA, USA). Samples were analysed using FlowJo software (v 10.5—Flow Jo LLC, Ashland, OR, USA).

### 2.4. DNA Methylation Analysis

From other whole blood samples, DNA was extracted using the Machery-Nagel NucleoSpin Blood kit according to the manufacturer’s instructions. DNA samples were relocated to the Australian Genome Research Facility (Melbourne, Australia) where bisulphite conversion and subsequent methylation quantification was conducted using the Illumina Methylation EPIC array platform according to the manufacturer’s instructions.

Epigenome-wide DNAm was assessed using the Illumina Infinium MethylationEPIC BeadChip (Illumina, Inc., San Diego, CA, USA), which interrogates >850,000 CpGs associated with over 24,000 genes. A standard protocol was used to process arrays [33]. To control for batch effects, samples were randomly allocated on microarrays. The bead chips were scanned by a BeadStation. We used Bioconductor packages IMA [34] and ComBat [35] for processing the methylation data and removal of batch-effect, respectively. The methylation level (β value) was determined for each CpG locus using the Methylation module of BeadStudio software. Beta values (β = $\frac{methylated}{methylated\;+\;unmethylated\;+\;c}$) indicate the proportions of methylated over the sum of methylated and unmethylated sites and c as a constant to prevent dividing by zero.

### 2.5. Prior Data to Conduct Focused Analyses

The Pregnancy and Childhood Epigenetics (PACE) consortium [29], in order to identify smoking-related CpG, used a population of 13 cohorts from the US and Europe that, with the same reproducible platform, measured CpG-specific DNAm across the epigenome in newborns. DNA isolated from cord blood samples was processed with the Illumina Infinium HumanMethylation450 (450K) BeadChip (Illumina), resulting in 6685 newborns participating in this meta-analysis. First, CpGs identified to be related to maternal smoking are taken from this EWAS, as performed by Joubert et al. [29]. In a second step, for further focus, we used the IoW birth cohort [30] to test which of the smoking-related CpGs are also associated with birth weight. The IoW birth cohort was established in the United Kingdom to study the natural history of asthma and allergic conditions prospectively. Details on study design, enrolment, and follow-up procedures are described in detail elsewhere [30].

### 2.6. Statistical Analysis

**Figure 1:**
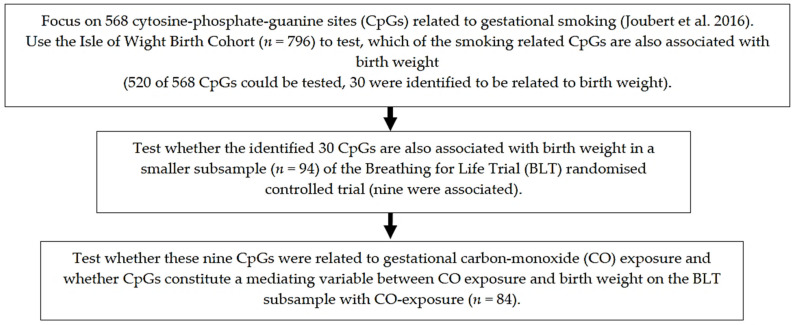
Flowchart of the analytical strategy identifying newborn DNA methylation sites that link gestational CO-exposure and offspring birth weight.

Analyses were conducted using SAS™ software, Version 9.4 of the SAS System for Windows, and Stata software (StataCorp. 2017. Stata Statistical Software: Release 15. College Station, TX, USA). In the IoW birth cohort, potential associations of DNAm sites with birth weight were screened using the ttScreening R package (v1.5, http://cran.r-project.org/web/packages/ttScreening/ (accessed on 1 February 2021)) [36]. This method removes non-informative CpGs in a course of 100 repetitions of a training-and-testing process with robust regressions. To estimate differential DNAm in the BLT cohort, we applied generalised linear models (GLM). A flow diagram of models and sample size is presented in Figure 1, showing the different steps in the analysis.

Chi-squared tests were performed to compare the distribution of demographic and obstetric descriptors among women with smoking and no smoking exposure during pregnancy. Models used confounders including maternal age, parity, and infant sex. To adjust for possible confounding in DNAm that might occur from cell composition in whole blood [37], we also selected seven cell types (lymphocytes TCD4 cells, lymphocytes TCD8 cells, Eosinophils, NK cells, neutrophils, B cells, and Monocytes) that were included as covariates in the linear regression. In the regression models, potential confounders were dropped from the full model, which includes all potential covariates one at a time, if the effects of interest in specific associations, i.e., CO-exposure and DNAm or DNAm and birth weight, did not change by more than 10% of the original values. DNAm sites were annotated based on data provided by Illumina. We applied the Bonferroni correction (*p* < 0.05/(the number of CpGs analysed)), to account for multiple testing in EWAS results.

Path analysis or structural equation modelling (SEM) [38,39] were conducted to assess whether the association between gestational CO-exposure and offspring birth weight was mediated by cord blood DNAm. SEM was used to estimate direct, indirect (mediated), and total associations between maternal CO-exposure and offspring birth weight. To reduce the number of covariates, we investigated whether covariates such as blood cell counts confounded associations assessed in the path analysis. We started with a full model including all reasonable paths, and then we dropped covariates one at a time until a parsimonious model was reached. We evaluated the goodness of fit (the empirical model is not statistically significantly different from the conceptional model) using the following criteria: (1) *p* > 0.05, (2) standardized root mean square residual < 0.08, (3) adjusted goodness of fit > 0.95, and (4) root mean square error of approximation < 0.05. All analyses were performed using the Statistical Analysis System (SAS 9.4, Cary, NC, USA).

## 3. Results

**Figure 2:**
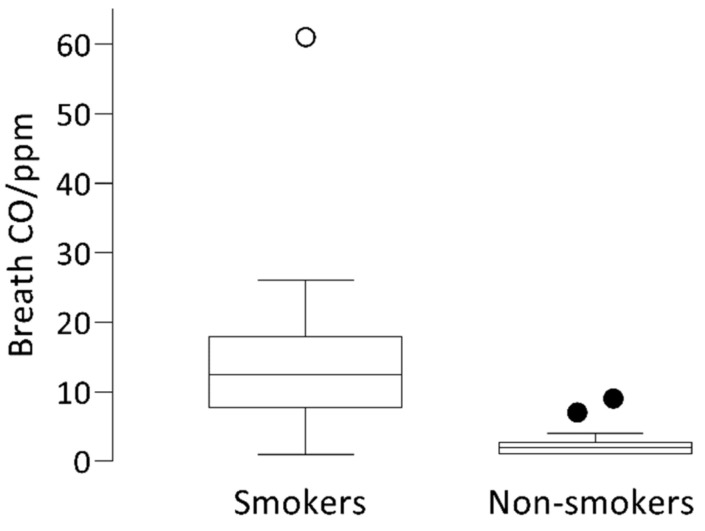
CO concentration in exhaled air in self-reported smokers (*n* = 11), and self-reported non-smokers (*n* = 83).

A total of 94 participants were included in the analyses; eCO was assessed in all participants. Eleven self-reported that they smoked during pregnancy (mean age 29.2 ± 5.2), and 83 self-reported not to have smoked during pregnancy (mean age 30 ± 4.8). Using an eCO level of 6 ppm as recommended by Gomez et al. [40] as the reference point to validate smoking abstinence, eleven participants (approximately 12%) had eCO levels greater than six ppm (Table 1). The mean eCO levels were 16.8 ± 16.1 ppm for self-reported smokers and 2.1 ± 1.2 ppm for self-reported non-smokers (*p* < 0.001) (Figure 2).

The average difference in birth weight between self-reported smokers and non-smokers was 209 g (*p* = 0.18). Using the eCO (>6 ppm) to discriminate between smokers and non-smokers, the average difference in birth weight was 429 g (*p* = 0.005). Infant sex differences were identified, such as higher birth weight among males. In female newborns, the average birth weight was 459 g lower in the eCO ≤ 6 ppm subgroup compared to >6 ppm (*p* = 0.03, Table 1).

Among those with higher eCO levels (>6 ppm), 81.8% of the mothers reported smoking (Sensitivity), whereas among those with lower eCO levels 97.6% stated no smoking (Specificity, Table 2). The agreement between self-reported smoking status and measurable CO levels was substantial (kappa = 0.79) (Table 2). However, two reportedly non-smoking participants had eCO levels higher than the reference point, and two reportedly smoking participants had eCO levels ≤ 6 ppm.

Of 568 DNAm sites related to gestational smoking described by Joubert et al. [29], 520 could be tested. We used the IoW birth cohort to reduce the number of potential smoking related DNAm sites to those that also were associated with birth weight. Of the screened 520 CpG site, 30 were identified to be related to birth weight. The methylation is expressed as beta values ranging between 0 and 1; multiplied by 100, this indicates the percent of methylation of a CpG. Then, in the BLT cohort, nine of these 30 CpGs were significantly associated with birth weight (Table 3), adjusted for maternal age, parity, and infant sex. Their effects on birth weight are expressed per percentage of methylation.

Focusing on the nine CpGs significantly associated with birth weight, we then tested whether self-reported smoking or exhaled CO measurements were linked with these CpGs. Only one CpG in the body of the *LMO7* gene showed a significant association with eCO levels, and none were associated with self-reported maternal smoking (Table 4).

**Figure 3:**
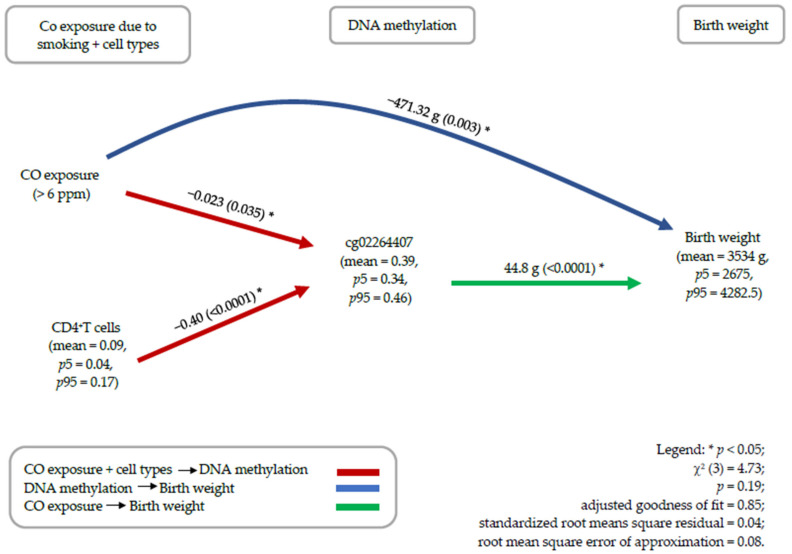
Path diagram—results from the structural equation model. Controlled for CO-exposure, CD4^+^ T cells, Eosinophils, B cells and gender. The effect of DNAm on birth weight in the path diagram is represented as the percentage methylation value (β × 100).

To further test whether this CpG site mediates the effect of smoking on birth weight, we conducted structural equation analyses (path analyses). Essential confounders were infant sex and the proportion of B cells, CD4 cells, and eosinophils in cord blood. Methylation at cg02264407 in the *LMO7* gene showed a mediating role between exhaled CO and birth weight (Table 5), controlling for confounders (Figure 3). CD4^+^ T cells, B cells, and eosinophils needed to be adjusted for, since the proportion of these cells also affected the methylation of cg02264407. Figure 3 shows a direct effect of CO-exposure on birth weight and an indirect effect of CO-exposure on birth weight via the CpG site cg02264407. *LMO7* methylation difference was most pronounced in CD4^+^ T cells negatively affecting its proportion in whole blood. Exposure to CO was associated with a 2.3% decrease in infant DNAm at the cg02264407 (*p* = 0.035). The average methylation at this site is 39%. For ease of interpretation and estimation, the effect of DNAm on birth weight on the path diagram is represented as the percentage methylation value (β × 100). For each 1% increase in methylation at this site, the birth weight increased by 44.8 g (*p* < 0.001). In other words, a 1% reduction of methylation of cg0226440 by eCO also resulted in a reduction of birth weight by 44.8 g.

## 4. Discussion

We measured variation in cord blood DNAm in 94 infants born to mothers with asthma. Using secondary analysis, an analysis focusing on 520 smoking related CpGs sites was used to identify that CpGs also associated with birth weight. We discovered 30 CpGs. Then, in the BLT cohort, we tested whether self-reported smoking status during pregnancy, and/or CO exhalation as a biomarker of current cigarette exposure, are statistically significantly associated with any of these 30 DNAm sites. We found nine CpGs sites (on nine genes), after Bonferroni correction, to be significantly associated with birth weight. Focusing on the effects of maternal smoking, the methylation of cg02264407 on the *LMO7* gene was significantly linked with exhaled CO measurements. Interestingly, none of the CpGs was associated with self-reported smoking. Finally, path analyses showed a direct effect of CO-exposure and an indirect effect of CO-exposure on birth weight via the CpG site cg02264407 on the *LMO7* gene. These results add to the developing literature demonstrating that maternal smoking during pregnancy can influence epigenetic modifications in early fetal/infant development [22,29,41,42], providing a likely mechanism linking them to altered gene function and perinatal health outcomes.

Assessing an epigenetic role in disease pathogenesis is of interest when investigating physical development in early childhood [22,43], in particular when related to early life exposure from tobacco smoke [44]. Gene expression modulation can be regulated by an epigenetic mechanism, including changes in DNAm, thus affecting fetal growth, and contributing to chronic disease development in later life. The influence of intrauterine exposure of tobacco smoke and their epigenetic changes have been reported for cord blood samples [45,46] but also later in childhood [29] and adulthood [47] demonstrating the impacts of maternal smoking during pregnancy on the offspring when DNAm persists independently of active smoking. Surprisingly, none of the CpGs in our sample were associated with self-reported smoking status; however, one CpG was significantly linked with exhaled CO measurements. Thus reporting bias (women may not reveal their smoking status during pregnancy) can affect the assessment of smoking, particular in a small sample [48]. Unbiased eCO measurement helped to detect significantly differently DNAm. Hence, to avoid a social desirability bias we decided to progress the analyses using the eCO as a measure of exposure [49]. The half-life of carbon monoxide ranges from five to six hours, and its elimination is related to minute ventilation, the full length of carbon monoxide exposure and the percentage of inspired oxygen [50]. This may explain why two participants who self-reported as smoking during pregnancy had eCO level lower than six ppm.

Figure 3 shows a direct effect of eCO on birth weight and an indirect effect via DNAm. Supporting pregnant women to quit smoking during pregnancy is the most significant intervention that can be established to decrease the risk of adverse birth outcomes; however, since there is an indirect effect, we may mitigate the negative effect on birth weight by blocking the indirect path. For instance, to deal with the adverse effect of maternal smoking, a recent study suggests that daily supplementation with vitamin C administered to pregnant women who continue to smoke tobacco may restore the effects of smoking-related changes caused for specific CpGs [51]. In addition, dietary interventions in adults have been shown to potentially improve DNAm changes caused by smoking exposures [52]. This could be an indication of a possible mechanism of intervening to reduce exposure effects after the exposure has already taken place, such as reinforcing the benefit of exclusive breastfeeding or potential dietary supplementation in offspring born to mothers who would not quit smoking during pregnancy [53,54,55,56]. However, more research is needed.

Our results are consistent with the hypothesis that tobacco smoke exposure in utero exerts direct biological influence on the developing infant through altering methylation of specific CpGs, in particular at *LMO7,* in turn affecting birth weight. Differential methylation of this gene related to maternal smoking was also observed in other EWAS in cord blood [57] and another study characterising smoking and methylation in adults [58]; however, it was not consistently replicated. *LMO7* is a multifunctional protein that can be found in several sites, mostly in the nucleus, plasm membrane, and at epithelial adherence junctions. Besides, it is broadly expressed in skeletal muscle and heart, with increased levels of expression in the lung [59,60,61]. Investigating the physio-pathological relevance of *LMO7*, gene ontology annotations reported connections between the *LMO7* gene and the biological process of regulation of cell adhesion (GO:0030155) and regulation of signalling (GO:0023051). A Reactome pathway [62] analysis showed significant upregulation of two pathways for Class I MHC mediated antigen processing & presentation (*p* < 0.05) and neddylation (*p* < 0.05). Albeit the latter hypothesis is controversial [63], deletion of the *LMO7* gene in mice resulted in an increased postnatal lethality due to a dramatic growth abnormality [64]. Moreover, it was reported to be imperative for appropriate skeletal muscle differentiation [65]. Despite these findings, the role of *LMO7* in human development has yet to be studied.

The small sample size of 94 pregnant mothers and their offspring is a limitation. Hence, we tried to focus on a limited number of DNAm sites that were detected in prior studies related to gestational smoking. Nevertheless, it is likely that other CpGs related to maternal smoking and offspring birth weight were not detected due to the reduced statistical power. In the present work, a potential limitation was that eCO measurements were not performed throughout pregnancy. The eCO measurement depended on the randomisation to the control or the intervention arm, which dictated a different regime of study visits. Consequently, we used only the baseline eCO measurement for both arms, it being unfeasible to use the data from attendance at other visits. A previous study indicated that differential methylation at some CpGs related to maternal smoking seems to require a sustained exposure to observe the effects on DNAm at birth [57]. Despite this limitation and the small sample size, we observed significant differences for the selected CpG and related CD4^+^ cells.

Strengths of our study include more objective measurement of CO as a marker of maternal smoking. Nevertheless, to avoid false-negative CO assessments, future studies should increase the density of CO measurement, since CO has a short half-life of five-six hours resulting in lower or non-detectable CO-levels in some participants. A second strength is the use of cord blood samples to assess DNAm as a likely mediator of adverse effects of in utero exposure to tobacco smoking on offspring birth weight without confounding from any post-birth exposures. Thirdly, even though we could only include participants for whom cord blood samples were available, the mode of delivery was not limited to vaginal birth. Thus, it is unlikely that only relatively healthier infants were included, excluding the possibility of underestimating the effects of smoking exposure during pregnancy. Fourthly, we compared associations of smoking related CpG sites with active tobacco use information collected through a self-administered questionnaire and eCO. The usual practice of analysing multiple exposures concurrently can lead to errors in explanation, as it is possible that the multicollinearity of exposures biases their assessment.

## 5. Conclusions

This is the first study to show that measurement of eCO between the 12th and 22nd week of pregnancy had inverse association with cord blood methylation of cg02264407 on the *LMO7* gene. In turn, a reduction of the methylation of cg02264407 was associated with lower birth weight. Our results are consistent with previous findings and demonstrate a potential epigenetic mediating effect with the consequence of in utero tobacco smoke exposure affecting fetal growth, showing a specific pathway via reducing the methylation of *LMO7*. Additional investigations to confirm these findings are recommended due to limited sample size. Finally, emerging approaches such as diet intervention hold the potential to prevent indirect adverse effect of maternal smoking on birth weight in vulnerable populations.

## Figures and Tables

**Table 1:** Characteristics of the participants for the association between umbilical cord blood DNAm and birth weight.

	Self-Reported Smoking			eCO > 6 ppm		
	Yes (*n* = 11)	No (*n* = 83)	*p*-Value	Yes (*n* = 11)	No (*n* = 83)	*p*-Value
Maternal age at childbirth, years (mean, SD)	29.2 (5.2)	30.0 (4.8)	0.659	29.9 (4.4)	29.9 (5.4)	0.999
Parity, nulliparous (%)	27.3%	54.9%	0.085	27.3%	54.9%	0.085
Gestational age at birth, weeks (mean, SD)	38.7 (0.4)	39.3 (1.3)	0.186	38.9 (1.3)	39.3 (1.4)	0.425
Infant sex, male (%)	63.6%	50.6%	0.416	54.50%	51.80%	0.864
Birth weight, (g) (mean, SD)	3328 (408.5)	3537 (487)	0.178	3134 (353)	3563 (475)	0.005
Birth weight females (g) (mean, SD)	3030 (345)	3452 (445)	0.074	3006 (303.5)	3465 (442)	0.030
Birth weight males (g) (mean, SD)	3499 (354)	3618 (516)	0.559	3240 (381)	3651 (491)	0.055
Socioeconomic Index for Areas (SEIFA), % (mean, SD)	4.1 (1.6)	4.9 (1.6)	0.139	4.3 (1.5)	4.9 (1.6)	0.276
Unemployment (%)	72.7%	30.0%	0.005	63.6%	31.3%	0.035
Caesarean birth (%)	36.4%	28.0%	0.568	27.3%	29.3%	0.888

**Table 2:** Agreement between self-reported smoking and measurable eCO.

	eCO > 6 ppm		eCO ≤ 6 ppm		Kappa
	No.	%	No.	%	0.79
Self-reported smoking	9	81.8	2	2.4	
No self-reported smoking	2	18.2	81	97.6	

**Table 3:** Methylation of specific cytosine by guanines (CpGs) from cord blood DNA associated with birth weight in the Breathing for Life Trial (BLT) subsample after Bonferroni correction for multiple testing.

CpG	Gene	Chromosome	Effect on Birth Weight in Gram per Percent Methylation ^i^	Standard Error (SE) of the Birth Weight Effect	*p*-Value
cg27434149	ANK3 *	10	−32.24	7.76	<0.0001
cg02264407	*LMO7*	13	49.13	13.37	0.0004
cg18444875	*OXR1*	8	−31.47	8.59	0.0004
cg22057874	*OSBPL6*	2	−55.21	18.35	0.0034
cg22902505	*PRDM8*	4	15.41	5.18	0.0038
cg06012804	HES1 *	3	29.28	12.69	0.0235
cg00169122	*ANKRD11*	16	−35.11	15.47	0.0257
cg07810039	*TGFB2*	1	−18.56	8.45	0.0308
cg09726279	*MYBBP1A*	17	74.06	34.36	0.0339
cg00624799	*ZNF710*	15	25.11	12.8	0.0529
cg07340025	ANKH *	5	−19.52	9.99	0.0541
cg12374579	*ASPSCR1*	17	28.74	14.81	0.0555
cg18183624	*IGF2BP1*	17	17.43	9.52	0.0707
cg04872675	TMEM119 *	12	20.54	11.43	0.0759
cg12160087	*CCDC64*	12	33.03	19.18	0.0886
cg25311470	*NRCAM*	7	45.57	28.11	0.1087
cg11043990	*RNF157*	17	−62.65	38.81	0.1102
cg00376553	*TSC22D4*	7	20.25	13.38	0.1337
cg23928512	*ASPSCR1*	17	19.44	12.94	0.1366
cg07638500	*MYLK*	3	16.40	12.55	0.1947
cg01668281	*CLDN14*	21	24.92	19.94	0.2148
cg18561976	*ICOS*	2	13.04	12.16	0.2867
cg14001239	*SVIL*	10	11.2	11.19	0.3198
cg24513387	LOC286083 *	8	22.94	23.01	0.3214
cg02973307	*KCTD15*	19	9.85	11.19	0.3813
cg13784312	*RAPGEF1*	9	−8.39	9.58	0.3835
cg02227813	*SAMD3*	6	−13.42	17.83	0.4538
cg07466788	*SLC16A3*	17	18.07	25.79	0.4853
cg26208507	CCND2 *	12	−6.93	12.86	0.5913
cg21611682	*LRP5*	11	0.62	23.13	0.9979

* Nearest gene. ^i^ Positive β difference indicates higher methylation and negative β lower methylation.

**Table 4:** Differential methylation in cord blood DNA in the BLT population associated with maternal smoking exposure during pregnancy.

				Self-Reporting Smoking			eCO		
Gene	Gene Group	Chr	CpG	Coef	SE	*p*-Value	Coef	SE	*p*-Value
*ANK3* P ^+^		10	cg27434149	0.023	0.020	0.246	0.036	0.020	0.081
*LMO7*	Body	13	cg02264407	−0.020	0.011	0.077	−0.023	0.011	0.041
*OXR1*	Body	8	cg18444875	0.023	0.017	0.177	0.030	0.017	0.087
*OSBPL6*	TSS200; Body	2	cg22057874	0.009	0.007	0.229	0.011	0.007	0.148
*PRDM8*	5′UTR; 5′UTR	4	cg22902505	−0.003	0.034	0.933	−0.033	0.035	0.347
*HES1* P ^+^		3	cg06012804	0.010	0.010	0.297	0.006	0.010	0.587
*ANKRD11*	5′UTR	16	cg00169122	−0.001	0.011	0.944	0.013	0.012	0.263
*TGFB2*	Body; Body	1	cg07810039	0.020	0.015	0.202	0.018	0.016	0.276
*MYBBP1A*	Body; Body	17	cg09726279	−0.007	0.005	0.141	−0.007	0.005	0.187

^+^ Nearest gene.

**Table 5:** Analytical path model showing the unstandardised coefficients for the associations of DNAm at the cg02264407 and CO-exposure with infant birth weight and *p* values (in parentheses).

	cg02264407	Exhaled Carbon Monoxide (eCO) > 6 ppm	Gender	B Cells	CD4^+^ T Cells	Eosinophils
Birth weight						
Direct	4476 (<0.001)	−368.07 (0.02)	−134.48 (0.16)	-	−388.66 (0.67)	-
Indirect	-	−103.25 (0.04)	-	678.4 (0.32)	−1791 (0.0003)	−586.33 (0.33)
Total	4476 (<0.001)	−471.32 (0.003)	−134.48 (0.16)	678.4 (0.32)	−2180 (0.08)	−586.33 (0.33)
cg02264407						
Direct	-	−0.023 (0.035)	-	0.15 (0.32)	−0.40 (<0.0001)	−0.13 (0.32)
Indirect	-	-	-	-	-	-
Total	-	−0.023 (0.035)	-	0.15 (0.32)	−0.40 (<0.0001)	−0.13 (0.32)

## Data Availability

The data presented in this study is available upon request to the corresponding author.
